# Odontological Society of Pennsylvania

**Published:** 1892-01

**Authors:** 


### ODONTOLOGICAL SOCIETY OF PENNSYLVANIA.

At the regular monthly meeting of the Odontological Society of Pennsylvania, held November 14, 1891, the president, Dr. Louis Jack, introduced the essayist of the evening, Dr. Rodrigues Ottolengui, of New York, who read his paper entitled, "A Study in the Regulation of Teeth."

(For Dr. Ottolengui's paper, see page 18.)

Dr. Ottolengui, on concluding the reading of the paper, referred to drawings on the board explaining them, and said,—

Another instance where a special clamp had to be devised for moving with a jack-screw was where Dr. Kingsley was regulating a set of teeth in which in the beginning there was a temporary molar removed, and during the progress of the case the bicuspid came down into position practically within the arch, and interfered with the work that was being carried on to such an extent that it became expedient to stop moving that tooth into position; moreover, the teeth on the opposite side of the jaw were very short, and it would have been impossible to put a plate in there which would have had any bearing upon the tooth, because the palatal cusps were too short. In this case I devised an instrument that did the work. An impression on each side of the mouth was taken separately and models made. This is to be remembered that, however short the tooth is in appearance above the gum, the whole tooth is present in the jaw. Consequently, with the plaster model before you, you can carve away the plaster till you have a fairly long tooth.

Now, around the bicuspid which was intended to be moved and around one on the opposite side I fitted bands made of pure gold burnished to the teeth. Then the two bands on one side were soldered together like the figure eight, and to that was fitted a little plate about three-quarters of an inch long and half an inch wide, which went over that part of the gum and was soldered to these bands, and on the plate was soldered a slot for one end of the jack-screw, and the band on the other side had soldered a little point for the reception of the jack-screw, and then the teeth were thrown out in about five days. So the jack-screw can only be used in extraordinary cases by some knowledge of obstacles and how to overcome them.

I wish to say one word about this other drawing (referring to board). I had intended bringing with me a set of models which were sent to me by another dentist.

They were evidently taken in some plastic material, and presented a drawn appearance around the teeth. He then had, seemingly, painted them with ordinary house paint and mounted them on an ordinary articulator, the plaster used to hold them being left as it was poured and white in color, and the whole thing was poorly made. He brought in the models and asked advice, which, of course, was refused without an opportunity to see the patient. Nothing could be told by these models, but when the patient came in I admit I was very much surprised to see what he had gained, because he had really made the jaw in six weeks quite presentable, and yet all the work he did was thrown away; and that is the reason why I wanted to notice the case.

This represents in a way (referring to board) what I wanted to demonstrate. The first glance would show that this tooth needs regulating, the tooth being within the arch, and in the six weeks he had made this tooth come into alignment, but an examination now of the jaw shows that the lower jaw closes imperfectly. The lateral incisor was nearer the correct position, and the other teeth should have been driven back instead of the upper incisor being driven out.

Dr. Kirk requested Dr. Ottolengui to go over again the line of reasoning which he adopted in the first part of his paper by which he determined whether he would widen the arch or move forward the central incisors.

*Dr. Ottolengui.*—That was a simple case. A close examination shows that not only are these teeth wrong, but at present the general effect is that it is a symmetrical jaw. We expect a fairly curved line in the jaw, and if these models will be opened and looked at in that way you will see what a marvellous difference there is between it finished and its first appearance, as before it was a straight jaw, being a straight line on each side. The lower jaw is retracted in reality. The incisors turned up the lip in a way that made a very ugly appearance. (Illustrated by a drawing on the black-board, showing the sides of the upper lip protruding noticeably by reason of the projecting teeth.)

Of course it could have been retracted, I suppose, by taking out the teeth, but as long as we had arranged to jump the bite, it was advantageous to widen the jaw, because if you attempt this operation without doing this, you cannot get good occlusion.

Dr. Roberts inquired how long he was in correcting the irregularity from the time he started until he put the retaining appliances in place.

*Dr. Ottolengui.*—There were just six weeks used in widening the arch, I think two weeks for correcting the irregularity in the front of the mouth, and three or four months for jumping the bite.

I might mention in that connection a reason that would account for the length of time it required. I made a retaining plate for the patient, and the first one was very comfortable; but the span was too far back, and afterwards I took an impression and made a new plate which would do the work, and she came to the office without this, and it required four or five weeks to get the plate from her, because it was more comfortable, and she would wear it. Unless I could have secured that, I suppose the bite would never have been jumped.

*Dr. Kirk.*—I am very much interested in this subject. At present I have four cases of this type of irregularity on hand. Two of them have been completed and two are in process of regulating now, and the feature of widening the arch in relation to this form of irregularity is, I think, quite interesting. I have examined the models more closely since asking the question, and I find there has been a gain in width at the cuspid teeth of nearly one-eighth of an inch. I should have adopted the plan of moving outward the central incisors first, because it would not have occurred to me to widen the arch as a preliminary. The jump of the bite is quite an important feature of the case. The lower jaw was retracted and held in an abnormal position by the irregularity of the superior central incisors. As soon as released the lower jaw took its normal forward position. The additional widening of the arch seems to have produced a more symmetrical form of the face than could have been had under any other method of treatment.

To my mind the apparent protrusion of the upper jaw was not an abnormal condition. In regard to moving outward the central incisors, the method described is quite ingenious and applies particularly to this case; but for four similar cases of my own I have adopted the plan, which I published some time ago, of using piano wire (indicates on the board), carrying the clasp around the molar, soldering on the outside of each clasp a little piece of gold tube, closing one end and leaving the other open; and then bending the piano wire to a U shape and letting it run into the tube sockets. These two clasps are united merely by a little silver plate which is used for the purpose of securing greater rigidity in the abutment.

I have used a modification of the same plan by putting tubing on the inside, with a slot cut in it and with buttons fastened on the free ends of the wire, carrying the wire inside of the arch, and tying the teeth to the spring thus formed on the palatal side for drawing in incisor teeth.

There is another plan which I have used for retracting teeth which is very satisfactory, and that is to swage a cap of thin platinum plate, about No. 31 or 33, over the teeth, slightly perforating the sides of the cap and cementing this on to the molars, and having a button or hook fastened on at the lingual or palatal part of the appliance. By such an arrangement you can use it satisfactorily with rubber bands for retracting the teeth. It is sometimes necessary to keep the teeth separated, and these caps open the bite sufficiently to allow that, and it is perfectly firm in its place. When I first used the cemented caps I found the cement did not adhere, but by making slight indentations around the edge of this swaged cap I was able to make it adhere nicely. These two methods have given me very satisfactory results.

Dr. Kirk, at the request of Dr. Ottolengui, described the proper way of tying a ligature to avoid slipping

Dr. Roberts stated that he had used a modification of the appliance of Dr. Kirk, with the exception that, instead of piano wire, he had used half-round gold wire and cut a thread on either end. The nut comes up solid against the lug. Tie the band down to the tooth first and screw up the nuts. The patient can do it with a little wrench. Dr. Roberts had been very successful in that way. In conclusion he stated that he was very thankful to know the proper method of tying ligatures as had been so clearly illustrated by Dr. Kirk.

*Dr. Guilford.*—We all feel very much indebted to Dr. Ottolengui for coming over from New York and reading us his very interesting paper. I would like to agree with him in all he said, but am sorry to say I cannot. One of the cardinal principles in regulating the teeth is simplicity. The doctor has made a very fine operation, but it seems to me he has taken the longest and most difficult way of accomplishing it. By an examination of the models that we have I see no reason in the world why, in the one case where he extracted the first bicuspid, he could not have done exactly the same thing in another. By extracting the first bicuspid in the diagram (referring to black-board) he could have drawn the lateral and cuspid back. The models show that there is room after that, but by spreading the arch, throwing the central back, and bringing the lateral in and jumping the bite he has done a great deal of unnecessary work, according to my view. It seems to me the best way is to take out one or both of the bicuspids and draw back one of the anterior teeth; that could very easily have been done.

There is one other point in regard to widening the arch. It is an error that I think a great many practitioners fall into. The widening of the arch is sometimes necessary in one jaw and sometimes in both, but it is a very tedious operation and in many cases a very difficult one. If you expand the upper arch you have to expand both. Dr. Kingsley, one of our earliest and best writers, says that, after all, the great desideratum in regulating teeth is to have a perfect occlusion,—to have as many teeth brought into use as possible in the mastication of food. I find, on examining the models, that the occlusion between the upper and lower teeth, so far as the bicuspid and molar are concerned, is not good, and the bicuspid on the other side does not touch those on the lower more than one-third as much as it did in the beginning. So, I think, he has lost in the expansion of the jaw in that case, in the matter of occlusion. Wherever expansion of the arch can be avoided and occlusion is secured, I believe it is better to leave it so.

The other point is that we can often simplify a case by the extraction of one or two teeth and get the same results that we would get by a more round-about way. In the first case I should simply have taken out the first bicuspid, and then by putting metal bands around the second molar and cuspid tooth, with little hooks properly placed, and by the aid of rubber bands, the teeth could be drawn back one at a time with very little difficulty; then taking the bands off the cuspid and putting the same on the lateral, drawing it back in the same way. It can be done, if the patient is not over seven years of age, in a comparatively short time. Pressing out the incisor tooth is also readily done, if needed, in a very simple manner.

I would like to take exception to some other things stated. He said that in taking the impressions for models he took them in plaster of Paris. There are some gentlemen who believe in plaster of Paris for all impressions. In going into the office of a dentist in this city, I was surprised to see on a table about five hundred pieces of plaster. He said he had been taking an impression of a case of irregularity. I have never had any difficulty in taking just as accurate an impression as I needed for cases of irregularity in modelling composition.

The essayist objects strongly to the use of the jack-screw. It can be abused,—there is no question about that. Where the patient is over twenty years of age, if we move a tooth too rapidly there is danger of devitalizing the pulp.

There is another point in regard to the rapid enlargement of the arch,—the danger of opening the suture where the two faces of the maxillary bones join each other. A friend of mine was exerting a great deal of pressure to expand the arch laterally; noticing that he was making unusual progress, he discovered that the sutures were opening, and he had to take off the appliance and let the case go for several months. The most powerful instrument we have for exerting pressure is the jack-screw. I use it wherever I can where great power is needed. Where less power is required I use the Coffin plate or metal springs, or something of that kind. When I want to work with rapidity, I cement platinum bands, with suitable lugs attached, to the teeth I wish to move, as well as those opposite them, and then exert pressure with the jack-screw. I see no objection to the proper use of the jack-screw. It is a very valuable instrument, and all it needs is to be used with care.

Dr. Deane asked Dr. Guilford if he did not think the bicuspids would regulate themselves, and that occlusion would be more perfect in a few years?

Dr. Guilford replied that he thought they would be better if they were farther back. I notice that one cusp in the upper jaw rests upon a corresponding cusp in the lower. Unless there is some pressure brought to throw that back, I don't see how it will ever take a proper position.

*Dr. Ottolengui.*—I think a great deal of the last speaker's criticism was due to the fact that he didn't hear all my paper or understand it. I certainly said nothing against the jack-screw, for I never have widened a jaw with anything else, consequently it would be impossible for me to speak against it. What I did speak of was certain obstacles in the use of the jack-screw, but it can be used intelligently, and I endeavored to point out some of those features.

I am glad that the last question was asked in regard to the possibility of jaws changing. Dr. Guilford raised the point that he could not see how that would correct itself without pressure. The pressure is produced in mastication. It is one of the agreeable things that, however irregular the jaw may be, the occlusion is generally good, except in cases of extensive protrusion of one jaw or the other. The removal of one tooth will produce elongation of another tooth, until occlusion is reached. So it is the power of mastication which will remove these obstacles.

The doctor spoke of a case of widening where the effect required an abandonment for months. I spoke of one in my paper where the suture had opened. I have seen it in a number of cases, and I rather like it, and I have never seen any harm from it, neither has Dr. Kingsley in the extensive practice he has had. In the case I have spoken of, the lateral teeth were hidden behind the centrals and bicuspids with no room to pass through. The jaw was widened in the hope of getting room to send the lateral out. At the end of a week it was seen that separation was occurring between the central incisors, which indicated that the suture was opening. Examination along the median line with the finger showed that the suture had opened, and the pressure was continued until the amount of room desired was obtained. A fixture was then put in which would keep the jaw at the point widened and regular. The case was continued without any regard to the fact that the suture was opened, and in about three months new bone was deposited all along the line, and was held permanently in place.

Where the jaw widens without opening the suture, what may happen? The alveolar process is simply taken up. After wearing the plate for three months there will be a tendency for the process to return to its normal condition, whereas, if you open the suture and have a deposit of new bone between the separated portions, there is placed a permanent wedge there to keep the jaw from coming back.

A word about impression materials. I don't think it is ever necessary to break an impression into a thousand pieces. The more irregular the case, the more probability there is that the impression would break up, and the more necessity there is to take it in non-yielding material. It stands to reason that if the teeth, which stand in the arch irregularly, and the hard plaster will not come out, the wax will not; but if the plaster will fracture, you can afterwards take the pieces and put them together, and you have an accurate model.

I want to say a word about extraction of the tooth in this case. I can't say there was really protrusion of the upper lip. There was protrusion opposite the lateral incisors, so that when the mouth was at rest you could observe a disfigurement over these.

The corners of the mouth were drawn in, and that was the reason I concluded to draw the laterals in. That action reduced the lip. The extraction of a tooth would be, in my opinion, a most deplorable thing, for it involves a greatly-increased space. We very seldom realize how much space a tooth will make until we take it out, or how much wider this looks than the tooth did when it was there, and therefore I think widening of the arch was permissible and advisable, because it allowed jumping of the bite; it gave a wider arch and a more symmetrical one, and, to a certain extent, it relieved the crowded condition you find on that side.

A word about screws. I had one of those very trying cases this summer where a bicuspid erupted without the arch. There was room enough for its reduction by a slight change of the other teeth, and after a space was made it was merely required to have an instrument to bring it into place. It is quite true nature may have accomplished this unassisted, but it had remained in that crowded position for a year and a half, and, therefore, it seemed advisable to force it into position. I made a plate, putting a couple of hooks on it, and threw an elastic band around the tooth, and brought it into position in one week. Now, what was the harm in taking my elastic band, instead of working with a screw? Every one who makes a practice of using springs or screws instead of rubber will admit, when he looks over his cases, that it takes, it may be, five months to do that which can be done by continuous pressure in half the time. What may be lost by possible danger to the tooth by moving it rapidly is more than counterbalanced by the comfort of the patient, and in causing the appliance to be worn not more than half the usual time. I think the nerves of the patient are to be considered as much as anything else. In the appliance of Dr. Kirk's I cannot see any special use of the connecting-plate. I had a case within a week in which I was in a quandary whether to make such an appliance or not, and I was satisfied that a single band was not suitable and it would not hold, and I made an arrangement similar to that described and fitted it to the mouth. I asked Dr. Kingsley what I would gain by attaching them by a plate. He replied that he did not know; and neither do I. I don't see what can be gained. It only adds something for this clasp to do. The clasp would have to hold the plate up as well as itself. Where you can get two molars with anything like a knuckle on them, make a rubber wedge and fit that clasp to go to that point and keep them wedged until the appliance is placed, and as soon as it is pressed down you have a point that prevents that clasp from riding up. You can always force it up, because half-round wire is always wedge-shaped, and it won't slip.

*Dr. Truman.*—I appreciate the difficulty of discussing a case of this kind, when the patient is not present, to take into consideration all the features of it, but I do want to enter my protest against a practice I consider physiologically wrong in the regulation of teeth. I cannot understand how we can go on moving teeth continuously without general disturbance to the individual. I am thoroughly of the view that all regulation should be made intermittingly. Time should be allowed for the individual to recover from the nervous strain and for the formation of tissue. As Dr. Guilford has stated, I think, very truly, that there is a liability of the destruction or strangulation of the pulp, and there is increased danger in the irritation of the pericementum. Slight irritation is absolutely necessary, in my opinion, for the reformation of bone which must take place after the moving of the tooth to replace that carried away by absorption. Now, if you advance too rapidly, an inflammation is aroused which may end in the destruction of the tooth. Therefore I do not approve, as a rule, of bands or ligatures moving with a continuous pressure.

I would like, before the subject is closed, for Dr. Ottolengui to give his views on that point. It seems to me a very important one in the regulation of teeth.

*Dr. Ottolengui.*—If we can move the teeth with rapidity it does not seem to me physiologically wrong.

If any one else in the room was under the impression that I

believed in risking the health of the patient by rapid movement of the teeth; I am very much pleased to have an opportunity of answering that question. As Dr. Truman very truly says, it is a physiological fact that there is danger in the too rapid motion of the teeth; but the question arises, What is a too rapid motion? If a tooth can be moved in eight weeks, it is more of an injury to take fifteen weeks to do it. It is important for a practitioner who is moving teeth to be able to appreciate when a tooth is being moved too rapidly. I don't see that it is giving the patient rest with an appliance in the mouth that works slowly, but the entire stoppage of the pressure for a week or two weeks, when it is seen the teeth are moving too rapidly, is the kind of work which I prefer. It is, therefore, an advantage to make an appliance that the patient can put on or off himself.

*Dr. Kirk.*—A very interesting point has been raised by Dr. Guilford in connection with this case of Dr. Ottolengui's which I don't think has been sufficiently answered or discussed. It involves the principle on which the irregularity should be corrected. If the models show anything, they show in the first place malocclusion of the two jaws. The lower jaw occupies an abnormal position. It shows that the lower bite occupies at least the width of one tooth or half a tooth back of its normal place. Also that the cause of the malocclusion is the abnormal position of the superior central incisors. By the method Dr. Ottolengui has pursued he has brought the occlusion of the jaws back to their normal condition; he has done the very best that could have been done in that case, while if the irregularity had been treated as Dr. Guilford advocated,—viz., by the extraction of the bicuspid teeth,—he would not have restored the normal articulation which has so greatly improved the case treated by the method of Dr. Ottolengui.

Dr. Guilford spoke of a case of a little girl where the upper teeth protruded much. As the patient resided outside of the United States and could not remain here, some appliance had to be used that could be operated by her local dentist under instructions. After extracting the two first bicuspid teeth above, a Coffin plate was made covering the palate and all of the posterior teeth. On the buccal surface of this plate a half-round gold wire was embedded, passing around the arch and terminating in a threaded round wire. This engaged with a nut fitted into a slot in the rubber on the opposite side. The object was to draw the anterior teeth back,—six of them. In a few months the teeth were brought into proper position.

(The appliance was further described, and the doctor stated that the results were very satisfactory, although the work had been done by a local dentist and entirely by written instructions.)

Adjourned.

